# A Digitized Representation of the Modified Prandtl–Ishlinskii Hysteresis Model for Modeling and Compensating Piezoelectric Actuator Hysteresis

**DOI:** 10.3390/mi12080942

**Published:** 2021-08-10

**Authors:** Chao Zhou, Chen Feng, Yan Naing Aye, Wei Tech Ang

**Affiliations:** 1 School of Mechanical and Aerospace Engineering, Nanyang Technological University, Singapore 637459, Singapore; FENG0091@e.ntu.edu.sg (C.F.); wtang@ntu.edu.sg (W.T.A.); 2 Invenco Group Limited, Auckland 1023, New Zealand; yan9aye@gmail.com

**Keywords:** asymmetric hysteresis modeling and compensation, modified Prandtl–Ishlinskii (MPI) hysteresis model, piezoelectric stacked linear actuators, micromanipulation

## Abstract

Piezoelectric actuators are widely used in micromanipulation and miniature robots due to their rapid response and high repeatability. The piezoelectric actuators often have undesired hysteresis. The Prandtl–Ishlinskii (PI) hysteresis model is one of the most popular models for modeling and compensating the hysteresis behaviour. This paper presents an alternative digitized representation of the modified Prandtl–Ishlinskii with the dead-zone operators (MPI) hysteresis model to describe the asymmetric hysteresis behavior of piezoelectric actuators. Using a binary number with *n* digits to represent the classical Prandtl–Ishlinskii hysteresis model with *n* elementary operators, the inverse model can be easily constructed. A similar representation of the dead-zone operators is also described. With the proposed digitized representation, the model is more intuitive and the inversion calculation is avoided. An experiment with a piezoelectric stacked linear actuator is conducted to validate the proposed digitized MPI hysteresis model and it is shown that it has almost the same performance as compared to the classical representation.

## 1. Introduction

Piezoelectric actuators are widely used in micromanipulation applications due to their rapid response and high repeatability, for example, microsurgical robots [1,2], autofocus optical systems [3,4,5], precise fabrication [6,7,8] and other applications [9,10,11]. One of the biggest challenges while using the piezoelectric actuators in dynamic applications is to model and compensate for the undesired complex hysteresis.

Various methods have been proposed for modeling and compensating the hysteresis behavior. The existing methods can be classified into physics-based models and phenomenology-based models [12]. Physics-based models [13] are often derived on the basis of physical principles of certain material or system properties [14,15,16]. Physics-based models often require a deep understanding of the causes of hysteresis and are often specific to the related properties.

The phenomenology-based model can also be roughly divided into three main groups [17]: differential-based models, such as the Bouc-wen [18] model, the Duhem [19] model, the Dahl, and the LuGre [20] model; neural-network models, such as the back propagation neural network based model [21], the gated recurrent unit based model [22], the neural network adaptive control method [23] and etc; operator-based models, such as the Preisach [24] model, the Krasnosel’skii–Pokrovskii(KP) [25] model, the Maxwell-slip [26] model, the Prandtl–Ishlinskii(PI) [27] model, and their variations.

The PI hysteresis model is one of the most popular models because of its simple implementation and analytical inversion [28]. Ang [29] concluded the relationship between the rate and the operators and modified the PI hysteresis model to model the rate-dependent hysteresis behavior. Tan [30] extended the PI hysteresis model to compensate for the ill-conditioned hysteresis behavior with a negative gradient. Mohammad [31] proposed a nonlinear play operator for modeling the asymmetric hysteresis of the Shape Memory Alloy (SMA) operators. Kuhnen [32] proposed to use the dead-zone operators for modeling the memory-free asymmetric hysteresis behavior.

In the above literature, the design procedure generally consists of parameters identification of the description model and the construction of the inverse model as a desired compensator. The mathematical complexity of the identification and inversion problem depends on the selected modeling approaches [33]. The amount of calculation may increase significantly with an increase in the number of elementary operators.

In this paper, we present an alternative digitized representation with which the inverse model can be easily constructed to compensate for the hysteresis behavior. The inversion calculation is avoided. The proposed representation is also more intuitive due to the geometric meanings of the parameters of the operators.

The paper is organized as follows: Section 2 describes the classical representation from which the proposed representation can be derived. Section 3 gives the experimental results with discussion. Section 4 covers the conclusion.

## 2. Hysteresis Mathematical Model

This section describes the classical representation and an alternative digitized representation of the classical PI hysteresis model. A similar representation of the dead-zone operators is also described, the construction of the inverse model with the proposed representation is also presented.

### 2.1. Classical PI Hysteresis Model

**Figure 1:**
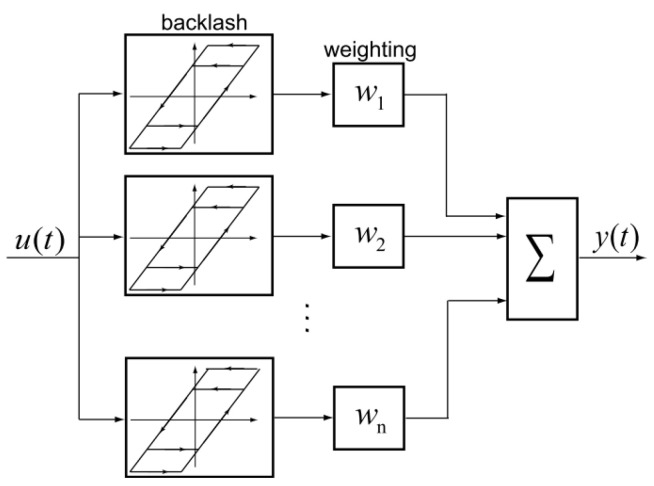
Working principles of the PI hysteresis model.

The classical PI hysteresis model has a series of elementary operators, the system output can be obtained by a weighted sum of n elementary operators with different thresholds. As shown in Figure 1, the working principles of the classical representation of the PI hysteresis model can be represented using the following equations:

(1) $$y\left(0\right)=y_{0}$$

(2) $$y\left(t\right)=\sum_{i=1}^{n}w_{i}\cdot max\left\{u\left(t\right)-r_{i},min\left\{u\left(t\right)+r_{i},y_{ei}\left(t-T\right)\right\}\right\}$$

where $w_{i}$ is the weight corresponding to the *i*th elementary operator, $r_{i}$ is the threshold of the *i*th elementary operator, *n* is the number of the elementary operators and $y_{ei}$ is the output of the *i*th elementary operator.

**Figure 2:**
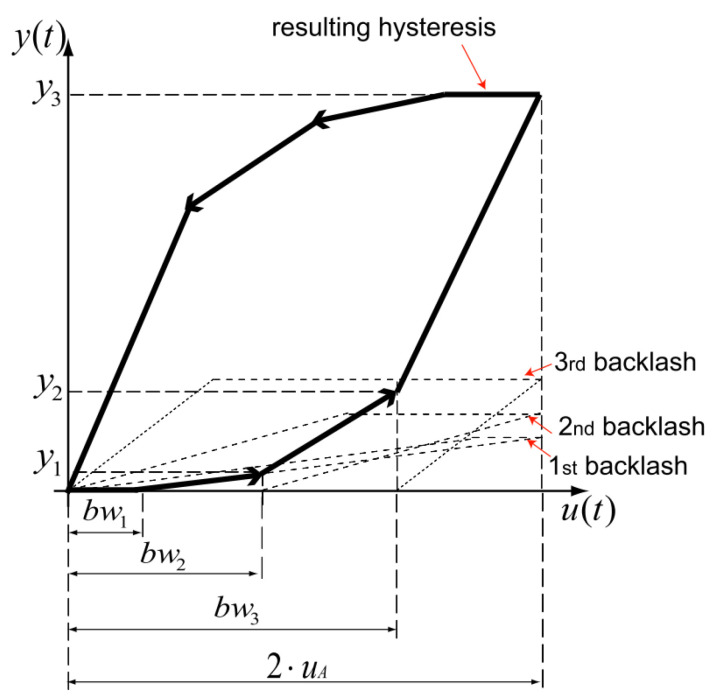
Example of (shifted) hysteresis obtained with three elementary operators.

When the parameter $r_{i}$ and $w_{i}$ are identified, the hysteresis curve model in the (u,y) plane is shifted so that it is in the positive section of the plane. An example with three elementary operators is shown in Figure 2, $bw_{i}=2r_{i}$ is the input value $x_{i}$ of turning point where the slope of the hysteresis curve is changed. The $k_{th}$ output value $y_{k}$ can be formulated as follows [34]:

(3) $$y_{k}=\sum_{i=1}^{k}\left(bw_{k+1}-bw_{i}\right)\cdot w_{i}$$

The Figure 2 can also be regarded as a series of 4 segments. Let $\left(\Delta x_{i},\Delta y_{i}\right)$ by the input and output value of each of the *n* segments, the value of the $\Delta x_{i}$ is the change of $bw_{i}$ while the value of the $\Delta y_{i}$ is the change of $y_{k}$. The slope $grad_{k}$ of the *k*th segment can be calculated using the following equation:

(4) $$grad_{1}=0;$$

(5) $$grad_{k}=\sum_{i=1}^{k}w_{i};$$

As $bw_{i}=2r_{i}$, the value of $\Delta x_{i}$ can be calculated using the following equations:

(6) $$\Delta x_{1}=2\cdot r1;$$

(7) $$\Delta x_{i}=2\cdot \left(r_{i+1}-r_{i}\right);$$

(8) $$\Delta x_{n}=2\cdot \left(u_{A}-r_{n-1}\right);$$

The value of $\Delta y_{k}$ can then be obtained using the following equations:

(9) $$\Delta y_{1}=0;$$

(10) $$\Delta y_{k}=\left(\sum_{i=1}^{i}w\left(i\right)\right)\cdot \Delta x_{k}$$

The paired property $\left(\Delta x_{i},\Delta y_{i}\right)$ represents the change of input and output value of each segment of the hysteresis curve and is thus more intuitive and the parameters are easier to be identified. The value of the paired property can be derived from the parameters of the classical representation. With this paired property, an alternative digitized representation of the classical PI hysteresis model is described in the next section.

### 2.2. Digitized Classical PI Hysteresis Model

**Figure 3:**
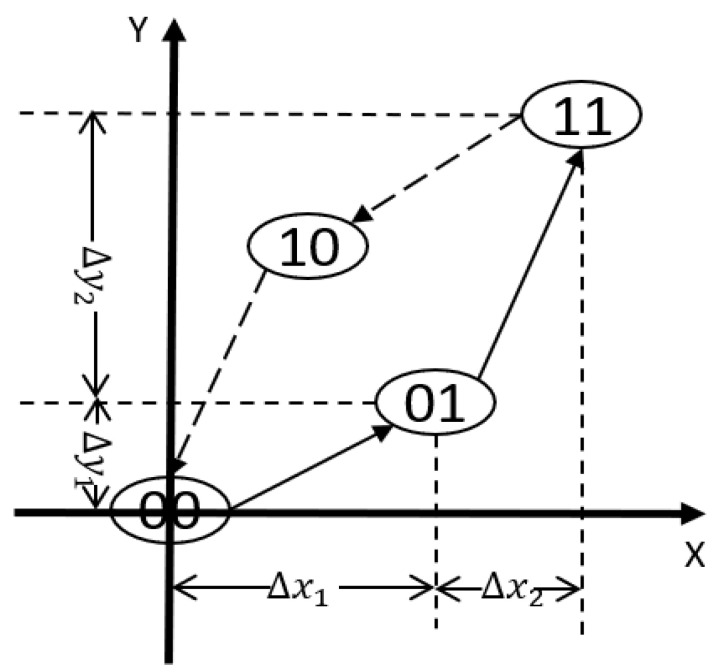
The paired properties of the digitized representation with two operators.

The state of the classical PI hysteresis model with n operators is represented with a binary number $B=b_{n}\ldots b_{2}b_{1}$ with *n* digits. Each digit of the binary number is a elementary operator with a binary state that can be either 0 or 1. Each elementary operator $b_{i}$ also has a paired property $\left(\Delta x_{i},\Delta y_{i}\right)$, where $\Delta x_{i}$ represents a change in the system input and $\Delta y_{i}$ represents a change in the system output. An example with two operators is shown in Figure 3. With an increase in the number of operators, the system output and input can be approximated to be a linear relationship within each operator. The paired properties *H* of a PI hysteresis model with n elementary operators can be expressed in one equation:

(11) $$H=\left[\begin{array}{ccc} \Delta x_{1} & \cdots & \Delta x_{n}\\ \Delta y_{1} & \cdots & \Delta y_{n} \end{array}\right]$$

The model also has a paired property $\left(X_{B},Y_{B}\right)$ that corresponds to its state. The state of the system always changes to either one of its two neighbors: upper state or lower state. Let $b_{1}$ be the least significant digit and $b_{n}$ be the most significant digit, the change of the state is always from the least significant digit. Let the least significant digit with a binary state of 0 at the current state be the *A* digit, let the least significant digit with a binary state of 1 at the current state be the *Z* digit.

When the state of the system changes to its upper neighbor, the binary state of the $b_{A}$ digit is changed from 0 to 1, the paired property of the system is increased by $\left(\Delta x_{A},\Delta y_{A}\right)$. Denote $X_{B_{t}}$, $Y_{B_{t}}$ be the system input and output of the current state and $X_{B_{t+1}}$, $Y_{B_{t+1}}$ be the system input and output after a change, the change to its upper neighbor can be shown in the following equations:

(12) $$X_{B_{t+1}}=X_{B_{t}}+\Delta x_{A}$$

(13) $$Y_{B_{t+1}}=Y_{B_{t}}+\Delta y_{A}$$

Similarly, when the state of the system changes to its lower neighbor, the binary state of the $b_{Z}$ digit is changed from 1 to 0, the paired property of the system is decreased by $\left(\Delta x_{Z},\Delta y_{Z}\right)$. Denote $X_{B_{t}}$, $Y_{B_{t}}$ be the system input and output of the current state, and $X_{B_{t+1}}$, $Y_{B_{t+1}}$ be the system input and output after a change, the change to its lower neighbor can be shown in the following equations:

(14) $$X_{B_{t+1}}=X_{B_{t}}-\Delta x_{Z}$$

(15) $$Y_{B_{t+1}}=Y_{B_{t}}-\Delta y_{Z}$$

**Figure 4:**
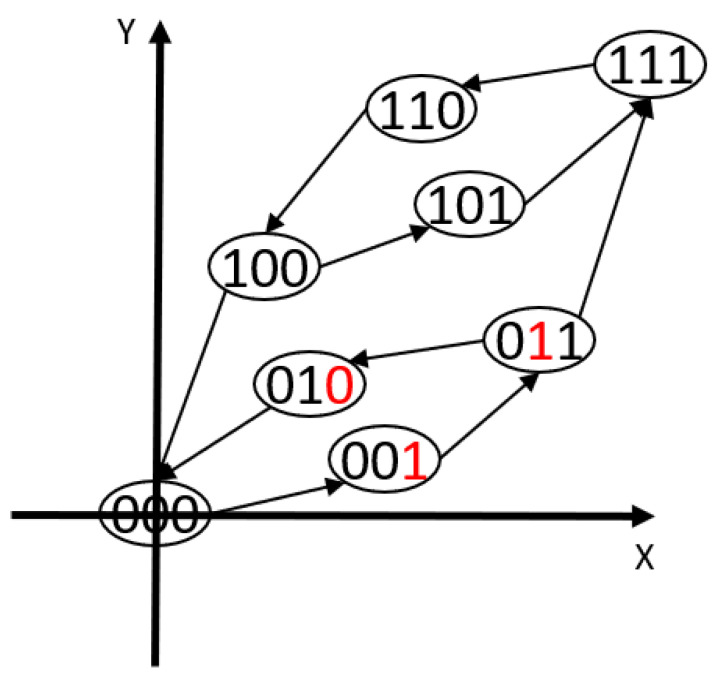
A digitized representation of the classical PI hysteresis model with 3 operators.

The traditional PI hysteresis model with n elementary operators has $2^{n}$ possible states. A simple example with 3 operators can be shown in Figure 4. The modeling and control accuracy may be improved with an increase in the number of operators.

When the system input is changed to *x* with the current state $B_{T}$, the system output can be calculated with the following procedure:

1. If the system input value, *x*, is greater than or equal to $X_{B_{T}}$ and less than $X_{B_{T}}+\Delta x_{A}$, the value of the system output y is calculated using (16).

2. If the system input value, *x*, is greater than $X_{B_{T}}+\Delta x_{A}$, the state of the system is changed to its upper neighbor by setting the $b_{A}$ digit from 0 to 1, the $\left(X_{B_{T}},Y_{B_{T}}\right)$ is updated using (12) and (13). The procedure is then repeated.

3. If the system input value, *x*, is less than $X_{B_{T}}$ and greater than $X_{B_{T}}-\Delta x_{Z}$, the value of the system output y is calculated using (17).

4. If the system input value, *x*, is is less than $X_{B_{T}}-\Delta x_{Z}$, the state of the system is changed to its lower neighbor by setting the $b_{Z}$ digit, the $\left(X_{B_{T}},Y_{B_{T}}\right)$ is updated using (14) and (15). The procedure is then repeated.

(16) $$y=Y_{B_{T}}+\frac{\Delta y_{A}}{\Delta x_{A}}\times \left(x-\Delta X_{B_{T}}\right)$$

(17) $$y=Y_{B_{T}}+\frac{\Delta y_{Z}}{\Delta x_{Z}}\times \left(x-\Delta X_{B_{T}}\right)$$

### 2.3. Digitized Dead-Zone Operators

**Figure 5:**
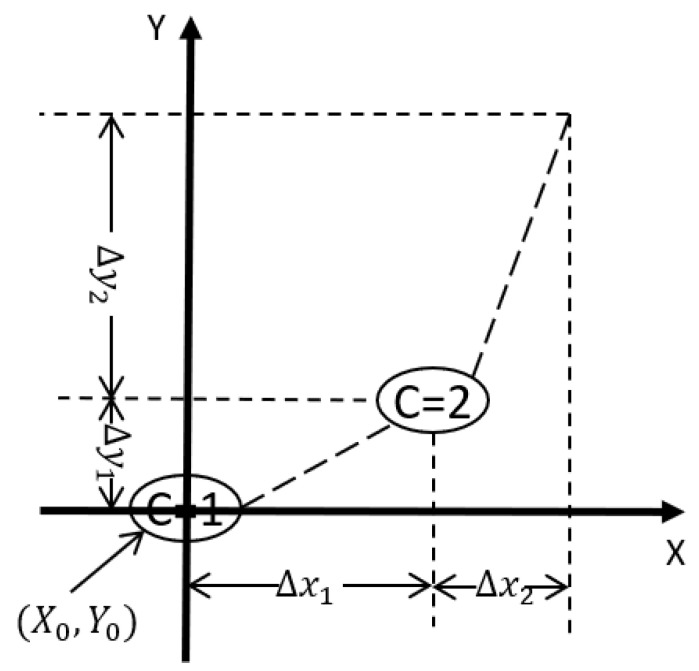
Paired properties of the dead-zone operators for extending the classical PI hysteresis model for asymmetric hysteresis behavior.

With the dead-zone operators, the modified PI hysteresis model can model the memory-free asymmetric hysteresis behavior. The state of the dead-zone operators can be represented with a number *C*. The *n* paired properties can be used to help describe the values of the *n* dead-zone operators as shown in (18). An example with two operators is shown in Figure 5.

(18) $$S=\left[\begin{array}{ccc} \Delta x_{1} & \cdots & \Delta x_{m}\\ \Delta y_{1} & \cdots & \Delta y_{m} \end{array}\right]$$

The model also has a paired property $\left(X_{C},Y_{C}\right)$ that corresponds to its state, the relationship between the paired property and the state of the model is described in the following equations:

(19) $$X_{C}=X_{0}+\sum_{i=1}^{C}\Delta X_{i};C=1,\ldots ,m$$

(20) $$Y_{C}=Y_{0}+\sum_{i=1}^{C}\Delta Y_{i};C=1,\ldots ,m$$

When the system input is changed to *x* with the current state *C*, the system output can be calculated with the following strategy:

1. If the system input value, *x*, is greater than or equal to $X_{C}$ and less than $X_{C}+\Delta x_{C}$, the value of the system output y is calculated using (21).
2. If the system input value, *x*, is greater than $X_{C}+\Delta x_{C}$ and *C* is less than *m*, the state of the system is changed to its upper neighbor by increasing *C*, the $\left(X_{C},Y_{C}\right)$ is updated by adding $\left(\Delta x_{C},\Delta y_{C}\right)$. The procedure is then repeated.
3. If the system input value, *x*, is less than $X_{C}$ and *C* is greater than 1, the state of the system is changed to its lower neighbor by decreasing *C*, the $\left(X_{C},Y_{C}\right)$ is updated by subtracting $\left(\Delta x_{C},\Delta y_{C}\right)$. The procedure is then repeated.

(21) $$y=Y_{C}+\frac{\Delta y_{C}}{\Delta x_{C}}\times \left(x-\Delta X_{C}\right)$$

### 2.4. Inverse Model

**Figure 6:**
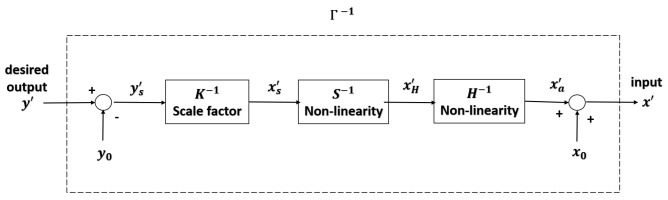
Block diagram of the inverse model as a feedforward controller.

The key idea of the parameters identification of the inverse model is to find the reflection of the resultant hysteresis curves about the $45^{\circ }$ line. A linear response is obtained by cascading the inverse hysteresis operator $\Gamma^{-1}$ as a feedfoward controller with the actual hysteresis. The proposed model for the inverse feedfoward controller is illustrated in Figure 6.

Unlike previous work, the parameters of the inverse model using the proposed representation can be found by simply exchanging Δx with Δy within the paired property, as shown in the following equations:

(22) $$H^{-1}=\left[\begin{array}{ccc} \Delta y_{1} & \cdots & \Delta y_{n}\\ \Delta x_{1} & \cdots & \Delta x_{n} \end{array}\right]$$

(23) $$S^{-1}=\left[\begin{array}{ccc} \Delta y_{1} & \cdots & \Delta y_{m}\\ \Delta x_{1} & \cdots & \Delta x_{m} \end{array}\right]$$

Due to its recursive nature, the calculation of the parameters identification of the inverse model does not change much with an increase in the number of the operators, while that of the modified PI hysteresis model is proportional to the number of the elementary operators. With the proposed digitized representation, the inversion calculation is avoided.

## 3. Experimental Results

In this section, the asymmetric hysteresis behavior of a piezoelectric stacked linear actuator, MPO-050100 (Nanofaktur, Stuttgart, Germany), is modeled using the classical representation as well as the proposed representation. Two experiments have been performed. The first experiment is to identify the parameters and to model the hysteresis behavior of the piezoelectric actuator. The second experiment is to test the performance of the proposed representation deploying the inverse model as a feedfoward compensator.

### 3.1. Experimental Setup

**Figure 7:**
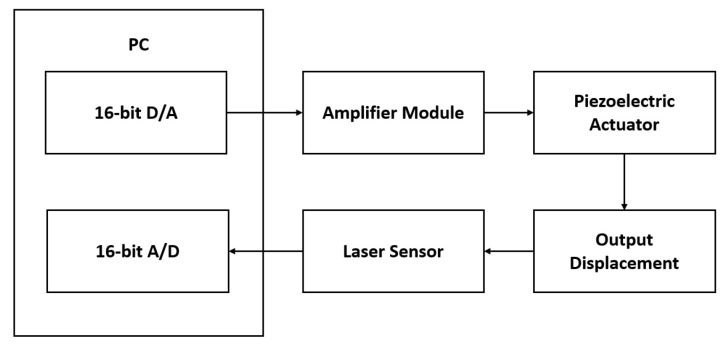
Experimental architecture.

**Figure 8:**
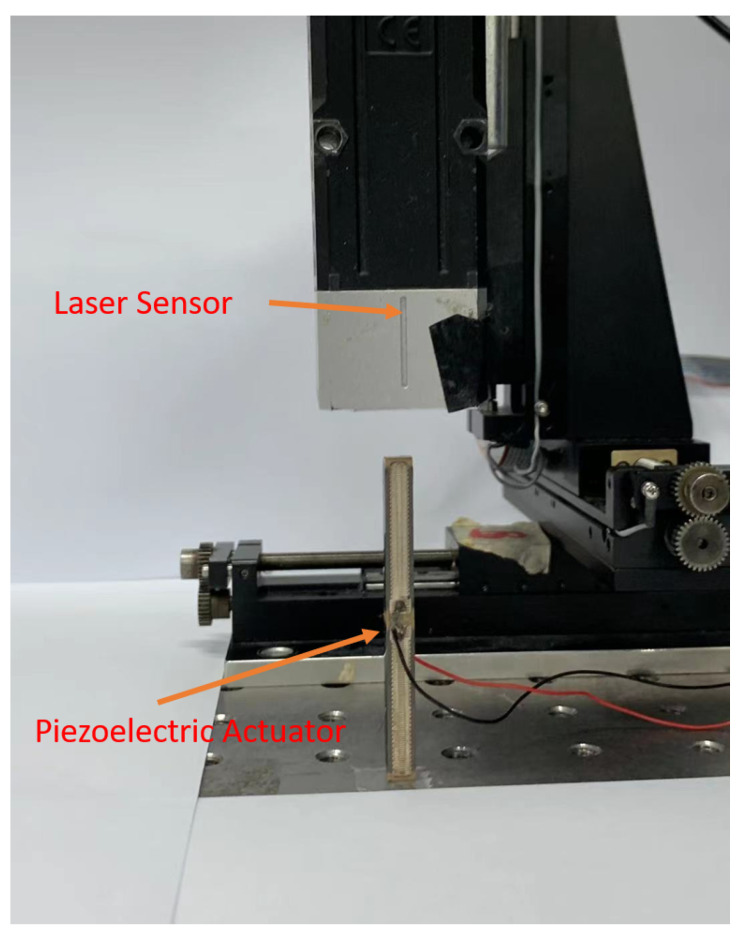
Experimental setup.

As seen from Figure 7, the input for the driver is a 10 V peak to peak, 1 Hz sinusoidal wave, is produced by a 16-bit D/A card. The input is passed to an amplifier, EMO-050100 (Nanofaktur, Stuttgart, Germany). The piezoelectric stacked linear actuator will deform, and the output displacement will be measured by a laser sensor, ZW-7010 (Omron, Kyoto, Japan) and is then converted to an analog signal which is received by a 16-bit A/D card. The experimental setup is shown in Figure 8.

### 3.2. Experimental Results

**Figure 9:**
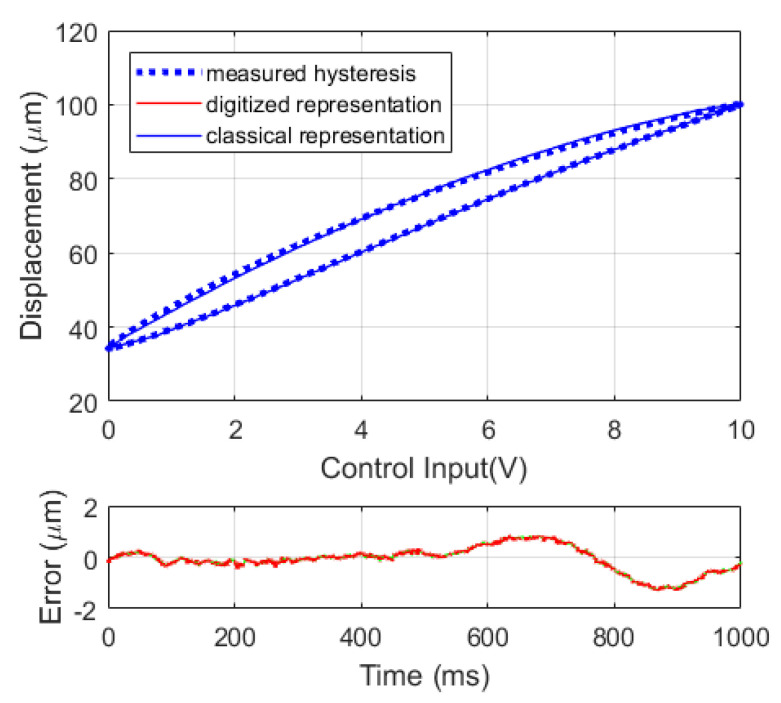
Modeling results using the classical representation and the digitized representation with dead-zone operators at 1 Hz, sinusoidal wave.

The hysteresis behavior of the piezoelectric stacked linear actuator under periodic control inputs is recorded. The parameters of the digitized representation and the classical representation of the modified PI model with the dead-zone operators are then identified with n=25, the identified parameters are used to model the hysteresis behavior of the piezoelectric stacked linear actuator. With the least square fitted models, the modeling results using the classical and the digitized representation with dead-zone operators are superimposed to the measured hysteresis behavior as shown in Figure 9. It is shown that the modeling results are almost the same using classical representation and the digitized representation. The root mean square error (RMSE) between the modeled hysteresis behavior and the measured hysteresis behavior are 0.50 μm using either of the two representation with a stroke of 66.14 μm. The modeling error is around 0.76% of its stroke. This proves that the proposed representation has almost the same performance compared to the classical representation while modeling the asymmetric actuator hysteresis.

**Figure 10:**
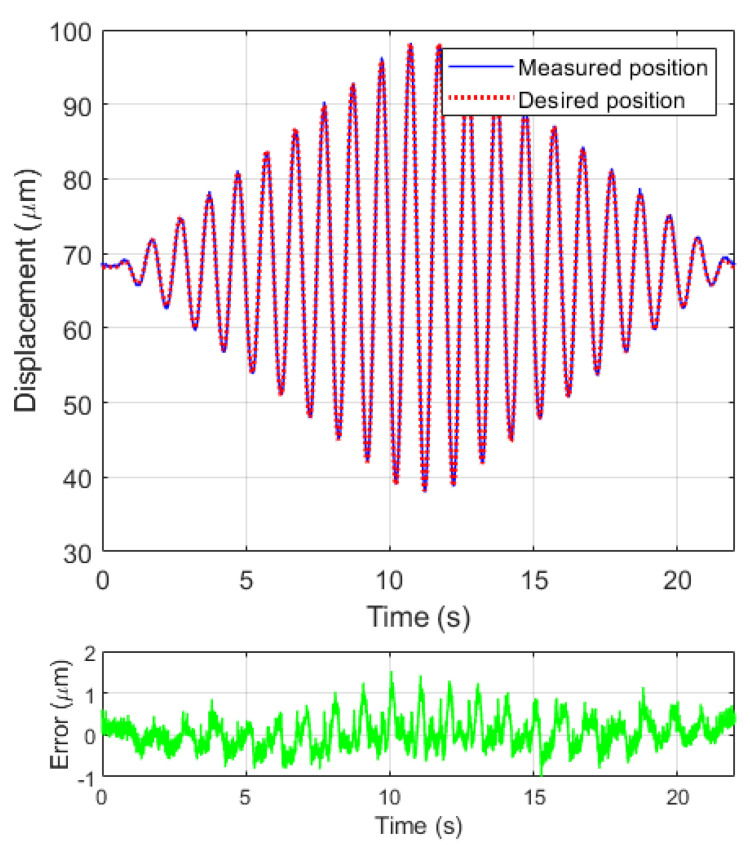
Compensation results at a changing amplitude sinusoidal wave.

The inverse model is constructed and is then applied to compensate for the hysteresis behavior of the system at a changing amplitude sinusoidal wave with $x_{d}\left(t\right)=A\times sin\left(2\times \pi \times t\right)+68.15$ (μm). The compensation results are shown in Figure 10. The RMSE between the measured compensated position and the desired position is 0.36 μm, with a maximum amplitude of 60 μm. The error is around 0.6%.

### 3.3. Discussion

Experimental results show that the proposed digitized representation performs almost the same as the classical representation while modeling the hysteresis behavior of the piezoelectric stacked linear actuator. This is because the digitized representation is derived from the classical representation and the two representation are equal mathematically. With n=25, the RMSE for modeling the piezoelectric actuator hysteresis is around 0.76%. The compensation experimental results with the RMSE of around 0.6% is even slightly better than the modeling results. This also proves that the proposed representation can compensate for the asymmetric actuator hysteresis. The description and compensation experimental results prove that the proposed digitized representation and its inverse model can model and compensate for the asymmetric asymmetric actuator hysteresis.

With the proposed digitized representation, the construction of the inverse model is less mathematically complicated and this improvement may be significant with a large value of *n*. The parameters of the proposed representation is also more intuitive with their geometric meanings; this may help with the parameters identification. The proposed representation is more intuitive and may be easier to be modified further.

## 4. Conclusions

The proposed digitized representation is an alternative to the classical representation. The inverse model can be easily constructed with the proposed representation and the inversion calculation is avoided. The proposed representation is also more intuitive. The proposed representation and its inverse model are validated with description and compensation experimental results.
